# Factors influencing family physicians’ willingness to prescribe opioid agonist therapy using virtual modalities: a qualitative analysis

**DOI:** 10.3389/fmed.2026.1769437

**Published:** 2026-03-12

**Authors:** Sarah Muñoz-Violant, Sarah Spencer, Ellie Gooderham, Shawna Narayan, Bohdan Nosyk, Rita K. McCracken, Lindsay Hedden

**Affiliations:** 1 Faculty of Health Sciences, Simon Fraser University, Burnaby, BC, Canada; 2 Faculty of Medicine, University of British Columbia, Vancouver, BC, Canada; 3 School of Medicine, Simon Fraser University, Surrey, BC, Canada

**Keywords:** opioid agonist therapy, opioid use disorder, prescribing, primary care, telehealth, virtual care

## Abstract

**Introduction:**

Although opioid agonist therapy (OAT) is the first-line treatment for Opioid Use Disorder (OUD), access, initiation, and retention rates remain low. An avenue to improve these rates is to enhance OAT delivery in primary care settings. As Canada continues to support hybrid models combining in-person and virtual care, evaluating the role of virtual care in supporting OAT prescribing and OUD management is necessary. This qualitative study explores the factors enabling and hindering family physicians’ willingness to prescribe OAT virtually.

**Methods:**

Utilizing maximum variation sampling, we conducted semi-structured interviews with 12 family physicians providing care to people with OUD in British Columbia, Canada. We audio-recorded, transcribed verbatim, coded, and thematically analyzed interview data using a hybrid inductive–deductive approach.

**Results:**

In this focused analysis, we identified themes across patient-, physician-, and health system-level factors in accordance with the Barriers and/or Facilitators to Primary Care Access for Individuals with Mental Health and/or Substance Use Issues framework. Patients’ individual characteristics, pre-existing patient-provider relationships, and physicians’ perceptions of patient stability each influenced prescribing decisions in virtual modalities. Providing care alongside an interdisciplinary team and access to province-wide centralized patient data systems or medical support networks also impacted physicians’ willingness to prescribe OAT virtually.

**Discussion:**

This study highlights the key factors influencing physicians’ willingness to prescribe OAT virtually and underscores the need for policy frameworks that empower providers to deliver equitable, patient-centred virtual care.

## 1 Introduction

Opioid Use Disorder (OUD) represents a significant global public health challenge that continues to require urgent policy attention from various sectors. Opioid agonist therapy (OAT) is the first-line, evidence-based treatment for OUD (1), reducing morbidity and mortality (2, 3) and improving individuals’ physical and mental health (4, 5). In Canada, OAT options include partial agonist therapies (i.e., buprenorphine/naloxone) and full agonist therapies (i.e., methadone, slow-release oral morphine) (6). The clinical stages of OAT include: (1) induction (or initiation) of medication; (2) stabilization, which aims to titrate the dose to a level that reduces cravings and withdrawal symptoms while monitoring and managing side effects; and (3) retention in treatment that extends to other determinants of health (7). While care for PWOUD is frequently centered on the provision of OAT, it also includes comprehensive and coordinated care for the treatment of co-occurring conditions.

Despite the benefits of OAT, the OUD cascade of care reveals consistently low engagement and high attrition rates across treatment stages. Only a small percentage of people with OUD (PWOUD) complete induction, and fewer than half of those achieve long-term retention (8–10). These challenges can be attributed to significant individual- and structural-level barriers, such as stigmatization, discrimination, inequitable healthcare access, and criminalization of drug use (11, 12). It is common, therefore, for PWOUD to go through cycles of engagement, disengagement, and re-engagement in treatment (13, 14). The risk of mortality during the first month following OAT treatment disengagement is higher than during the remaining time out of treatment, indicating that this pattern of repeatedly exposing PWOUD to periods of elevated mortality risk needs to be addressed (15).

Health researchers have continuously advocated for the development of treatment models that integrate OUD care into broader primary care to expand access to treatment, rather than receiving OUD care (particularly OAT) by a specialist or at the emergency department (16, 17). Primary care is equipped to provide comprehensive, stable, and longitudinal support, enhancing access to care and treatment retention (18, 19). In British Columbia (BC), family physicians who wish to provide OAT typically complete supplementary training in addiction medicine. To be authorized to prescribe full agonist therapies, family physicians who do not have a section 56(1) exemption – which allows practitioners to prescribe, transfer and provide controlled substances, under specific conditions to support continuity of care (20) – or who have not prescribed in more than three years are required to complete the Provincial Opioid Addiction Treatment Support Program certification by the BC Centre on Substance Use (21, 22). Providers who wish to provide partial agonist therapies or renew prescriptions initiated by other providers are not mandated to complete the training but are highly encouraged (23). Training requirements in BC are intentionally minimal to facilitate broader access to OAT, particularly in rural and remote communities (24). This approach also seeks to counteract barriers to OAT prescribing reported by physicians’ primary care settings, including insufficient funding and support, feeling overwhelmed, stigma, and skepticism about the effectiveness of OAT (25–27). Additionally, BC was the first province in Canada to implement task-shifting policies authorizing nurses to prescribe buprenorphine/naloxone and integrating pharmacists into patient care through dispensing and witnessing doses, which supports access and continuity of care and reduces reliance on physicians (28, 29).

OAT requires regular interactions with providers for prescription renewals and urine drug testing (UDT). Typically, OAT is provided via witnessed dosing and gradually transferred to take-home doses as PWOUD settle into their treatment (30). Nevertheless, because eligibility for take-home doses is often contingent on UDT, OAT requirements force continuous interactions with providers that disrupt patients’ lives and hinder adherence (31). A BC study found that weekly UDT only reduces the risk of OAT discontinuation by 4–6% in the first treatment year compared to no monitoring, which raises questions about the cost-effectiveness relative to patient burden (32). Consequently, medical experts have proposed the use of virtual care to reduce reliance on in-person visits while maintaining continuity of care, leading to the initiation of pilot interventions for OUD virtual care (33).

The COVID-19 pandemic accelerated the implementation of virtual care across all patient demographics, as public health measures shifted primary care to virtual modalities to support social distancing and reduce exposure risks for patients and providers. Studies have identified multiple benefits to delivering OAT via virtual care, including facilitating care accessibility, improving treatment retention and patient satisfaction, and participant flexibility and convenience (34–37). These results show the importance of empowering primary care physicians so that they are willing to prescribe OAT via virtual modalities. This qualitative study uses semi-structured interviews with family physicians to explore the factors enabling and hindering their willingness to prescribe OAT virtually.

## 2 Materials and methods

### 2.1 Study design and setting

As part of a convergent mixed-methods study, we conducted semi-structured qualitative interviews with family physicians (FPs) in BC, Canada. Refer to Hedden et al. (38) for a description of the full study protocol, study regions, and broader healthcare system context. Virtual care is defined in this study as synchronous interactions between patients and providers via telephone or videoconferencing. This focused analysis explores the perspectives of FPs prescribing OAT virtually. This manuscript is written in accordance with the Standards for Reporting Qualitative Research (39).

All interviews were conducted in BC. The larger research team is comprised of physicians who prescribe OAT, health policy and substance use researchers, people with living/lived experience with OUD, and knowledge users. The authors do not have lived experience with OUD. The positionality and professional experiences of the authors, including medical training, frontline work in low-barrier community settings, and involvement in addiction and health services research shaped the analytic process and interpretation of results.

### 2.2 Sampling and recruitment

We recruited participants using a purposeful maximum variation sampling strategy (40) to capture variance in FPs experiences and perspectives, including by gender, years in practice, practice model, and practice location. We included FPs with an active license to practice in Canada or who were enrolled in the Addiction Medicine Fellowship or Family Medicine Residency in British Columbia and who had experience with providing primary care for PWOUD.

We distributed study information via social media, study team members’ professional networks, and study participants (i.e., snowballing). We actively examined and adapted our recruitment methods throughout data collection to ensure the inclusion of demographic variability.

### 2.3 Data collection

We developed our physician interview guide (Supplementary material 1) in consultation with existing literature and members of the study team. The guide focused on participants’ experiences with and perspectives of providing virtual primary care to PWOUD. Specific questions addressed (1) accessibility and appropriateness of virtual modalities (video vs. phone visits) for PWOUD; (2) management of OUD and related comorbidities using virtual care; (3) impact of virtualisation on workflow, capacity and work satisfaction; and (4) any resources or supports upon which FPs relied for providing virtual care for PWOUD.

We conducted interviews via Zoom (Zoom Video Communications) or telephone, based on participant preference. Interviews were audio recorded and transcribed, except in one case where the participant requested not to be recorded. In this case, detailed notes were taken by the interviewers. All transcripts were verified by the interviewers.

### 2.4 Analysis

We followed a hybrid inductive-deductive thematic analysis approach (41) using NVivo 14 (42). This involved research team members independently familiarizing themselves with the transcripts and coding a subset of the data to inductively identify codes. The team then came together to discuss and compare codes and coding decisions and develop a harmonized codebook. We resolved disagreement through consensus, and tested our codebook and coding decisions over three physician transcripts, ensuring inter-coder reliability and trustworthiness (43, 44). Subsequently, three researchers (SMV, SN, and SS) coded all of the transcripts following the final harmonized codebook. We then grouped codes into themes, which our study team reviewed and refined through discussion.

These emergent themes align with Ross et al.’s (45) framework – Barriers and Facilitators to Primary Care Access for Individuals with Mental Health and/or Substance Use Issues – which we employed *post-hoc* to report our results. This framework proposes three levels of factors which influence primary care access: client-level; service provider-level; and health system-level.

### 2.5 Ethical considerations

We obtained ethical approval for this research from the Behavioural Research Ethics Boards at Simon Fraser University, the University of British Columbia, and the University of Victoria through the harmonized platform of Research Ethics BC.

The research team contacted interested prospective participants, provided them with information about the study and the consent documents. Individuals were requested to read through the study documentation, provide their written consent, and return it to the research team via email in advance of their interview. Data was de-identified and direct quotes are reported through unique participant IDs to uphold confidentiality.

## 3 Results

We conducted interviews with 12 FPs. The majority of participants practiced in urban areas of BC (*n =* 9, 75%), were women (*n =* 10, 83%), and had an average of 12 years in primary care practice. All participants had experience delivering virtual care. Participant characteristics are shown in Table 1. Key themes are presented in Table 2 and detailed below.

**Table 1:** Participant characteristics [*n* (%)].

Characteristics	Family physicians (*N =* 12)
Sex/Gender^a^	
Female/Woman	10 (83.3%)
Male/Man	2 (16.7%)
Addiction Training^b^	
None	3 (25%)
Provincial Opioid Addiction Treatment Support Program	6 (50%)
Addiction Medicine Fellowship	4 (33.3%)
Other	1 (8.3%)
Years in practice [mean (SD)]	
Mean	12.1 (10.0)
Hospital Privileges	
Yes	10 (83.3%)
No	2 (16.7%)
Community Size^c^	
Rural / Small Urban	3 (25%)
Urban	9 (75%)
Main Practice Setting	
Community-Based Practice	10 (83.3%)
Addiction Medicine Clinics	2 (16.7%)
Experience with Virtual Care	
None	0 (0.0%)
Phone Only	2 (16.7%)
Video Only	0 (0.0%)
Video and Phone	10 (83.3%)

^a^Gender was asked as an open-ended question; we group sex and gender here to reflect participants responses using a mix of gender and sex-based language. ^b^Participants may hold multiple addiction medicine certifications and thus be counted in multiple categories. ^c^Rural and small urba*n =* <99,000; Urban >100,000.

**Table 2:** Overview of themes.

Themes derived from Ross et al. (45)		Sub-themes identified during analysis
1.	Patient-Level Factors	OAT Stage Comorbidities OAT Medication Type Environmental triggers
2.	Physician-Level Factors	Pre-exiting longitudinal relationships Perceptions of patient stability
3.	Health System-Level Factors	Health system-wide electronic records Communication with interdisciplinary teams Specialized medical support networks

### 3.1 Patient-level factors

Within patient-level factors, themes about patient characteristics emerged, including OAT stage, comorbidities, OAT medication type, and environmental triggers. Participants expressed that a one-size-fits-all approach was inappropriate when deciding the suitability of virtual care for their patients with OUD. Rather, an individualized assessment was preferred with treatment tailored to patients’ unique circumstances and needs.

Individual patients’ OAT stage (i.e., induction, stabilization, or retention) greatly influenced the willingness physicians reported for prescribing OAT virtually. Generally, physicians were not willing to initiate OAT virtually:

…*I don’t see an induction with somebody you don’t know going well virtually. Yeah, it’s hard with patients you don’t know well. I think that has to be in person. They’ve got to show the commitment to show up, too. And that’s a little higher in person than it is on the phone – that commitment.* (FPOUD02)

However, when patients had discontinued OAT and were interested in re-starting, physicians were more open to virtual OAT prescribing: “*Because they have already done it [OAT induction] once, it’s not all that difficult on the phone*” (FPOUD02).

*…initiating [OAT] virtually on a patient that I know well, who has been off of their medication and wanting a restart. […] Those patients, I don’t have too much issue starting OAT over the phone because it’s kind of more like a restart.* (FPOUD11)

Our participants indicated that virtual care was particularly useful for gradual adjustments (titration) of OAT dosages to minimize opioid withdrawal and maximize therapeutic effects, as it allowed for closer monitoring in the form of consistent check-ins with patients:

*…one thing that virtual care has been really helpful for is, let’s say there’s someone that we’re really trying to titrate, like maybe they were recently on a high dose and then it was reduced, or they’re going to detox in a week, and we need to get them to a good dose fast. Pre-virtual care, you would say, ‘Oh, yeah, come in every three days for an increase.’ Or ‘If you’re on methadone or Kadian, come in every day.’ But now with the virtual care, I’ll say, ‘Okay, I’m going to call you tomorrow to go up,’ if it’s Kadian or whatever. So, I think that is actually really helpful. Because it does let you direct the follow-up a little bit more. Because if it’s in person, the person, you can’t make them come in the next day.* (FPOUD06)

FPs indicated that they assessed individual patients and their circumstances when determining whether delivering OUD care virtually was appropriate. Some participants reported being less willing to prescribe OAT virtually when patients presented with comorbidities that aggravated the overall clinical picture, preferring to rely on in-person care: “*So for patients that are more complicated, especially if they have [comorbid] complex chronic disease or there’s mental health or like cognitive issues, then those are the people that I definitely need to see in person*” (FPOUD03). Other participants utilized a hybrid model of care, where FPs employed virtual appointments to determine the need for and frequency of in-person visits:

*It depends on the comorbidity […] a lot of my patients have mental health stuff that we’re working on together, because… it plays into their [opioid] use disorder. […] And then if they’re doing poorly, again, you’re recalling them to see them in person more, which is a little more supportive. Or, like, one of my patients that I see for gender-affirming care in conjunction with her OAT, we try to do phone. But occasionally it’s nice to see her in person just for more of that counselling and sort of interpersonal connection piece.* (FPOUD10)

Some OAT medications (e.g., methadone) require more intensive monitoring than others (e.g., buprenorphine/naloxone). Participants reported that the type of medication they choose to prescribe could inform their willingness to prescribe virtually:

*I’m more flexible […] with my Suboxone patients. We know that Suboxone is a generally safer medication, with fewer concerns around diversion […] I typically am okay with seeing them in person every three to four months if they’re someone who I’ve been able to assess virtually in the interim. Whereas my methadone and Kadian patients […] I’ll typically want to see in person as much as possible*. (FPOUD11)

This is particularly true for medications that include supervised dispensation by a healthcare provider, as this can determine the frequency of in-person interactions that FPs require:

*The fentanyl patch patients – again, I’m actually a little bit more flexible with them because I know that they’re seeing our nursing team or pharmacy team three times a week for their patch changes, and that clinician will relay to me if they have any concerns over how the patient is looking or acting. So, because those patients kind of have built-in follow-up from the healthcare or allied healthcare provider regularly, I’m okay with assessing them however they want to be assessed.* (FPOUD11)

Finally, physicians reported that community areas with high rates of active street-based substance use can be a challenge for patients in recovery who previously frequented those environments. Many patients choose to leave those areas to avoid being around active substance use. Recognizing these situations, physicians were generally more flexible in prescribing OAT virtually, viewing it as a way to support their patient’s recovery journey.

*Or someone who's gotten out of the [neighborhood], ‘I really don't want to go back there, it's a huge trigger for me,’ and then we can just titrate them over the phone without exposing them to the potential harm and triggers that they associate in the [neighborhood].* (FPOUD07)

### 3.2 Physician-level factors

Within physician-level factors, two themes emerged: pre-existing longitudinal relationships and perceptions of patient stability. Participants noted that pre-existing relationships provide them with necessary context and an added layer of confidence in their clinical decision making when providing OUD care virtually. These clinical relationships are often built upon months or even years of in-person interactions between patients and either their physicians or the clinic.

… *there are people that I’ve literally known for 15 years, have been on OAT in that clinic. […] It makes it way easier. We had a group that a physician left quite suddenly […] So we took on about 100 patients. And it was really hard because to see them [virtually] was very, very difficult. And we had never met them before, right? So they may have been on methadone for several years under this other provider. But now we’re taking over. And that caused a lot of stress in the provider group, saying, I have no idea if I’m making good choices or good care, you know, talking to this person on the phone. And so I think that the people that we knew, [virtual care] felt like an okay transition. And then the people that were newer, [virtual care] felt really…a lot of us [providers] felt uneasy, really uncertain*. (FPOUD09)

A longitudinal relationship between a patient and a team or clinic, rather than with a single physician, can also support decisions to provide OAT virtually, as it allows them to access the patient’s history: “*In our practice […] you can look back at their [UDT results] for the last five years […]. I feel very comfortable starting people over the phone based on just the history*” (FPOUD06). Conversely, when access to a patient’s chart does not provide adequate or sufficient information, in-person visits may still be needed:

*I think that having access to the patient’s chart and all the family doctor’s prior notes […] is really valuable to me […] If you’re talking to somebody on the phone and [the chart is] not clear, just bring them in. And so I do that a lot. […] maybe it results in more visits because there are more times when I need to say, ‘Okay, we need to do a follow-up, I need to see you in person’. And maybe their family doctor could have said, ‘Oh, this is your old whatever acting up again’, and they wouldn’t have needed that.* (FPOUD04)

Physicians indicated that they relied heavily on their perception of a patient’s stability to make the decision about whether to offer OAT prescriptions virtually: “*a stable patient who is on long term OAT, who might be working or, you know, have stability in their lives, a phone call follow-up once a month, you know, with in-person every couple of months, is totally sufficient*” (FPOUD13). Participants varied, however, in what indicators (e.g., housing, employment) they relied on to assess patient stability when making this decision. Interestingly, the introduction of virtual care impacted the way some participants assessed patient stability to include indicators in broader aspects of the patient’s life:

…*we used to be so focused on the urine drug screen result as a primary marker of success of care […] and stability. [The COVID-19 pandemic] really did force me to shift to looking at what I think of as multiple markers of stability. So, how do they sound on the phone? Are they answering the phone, because their phone has minutes, regularly? Are they able to be pleasant and polite and appropriate in our conversations? Do they attend when I ask them to in person? Do they come for urines and such? Have they remained housed? Are they employed? These kinds of things. So you really do look at more of the full spectrum of their picture, as opposed to just what did their urine show.* (FPOUD10)

Nevertheless, in cases where physicians did not perceive stability, they tended to adopt a harm reduction approach to their OUD care. That is, participants expressed a willingness to meet individuals where they are in their recovery journey and minimize barriers to care: “*[I] really try to reduce the barriers as much as I can.*” (FPOUD06). FPs indicated that their priority was to keep patients engaged in treatment and connected with the healthcare system: “*I do not think I have any patients right now that I’ve never seen in person. And I certainly would [have only virtually-seen patients], if that was all that they were able to do to keep them on meds and treatment and engaged*” (FPOUD10).

### 3.3 Health system-level factors

Within health system-level factors, themes about health system-wide electronic records, communication with interdisciplinary teams, and specialized medical support networks emerged. Health system-wide electronic records such as PharmaNet – a secure network that connects all pharmacies to a centralized database to keep track of prescription dispensation in BC (46) – and CareConnect – a provincial Electronic Health Record (47) – allow physicians to monitor patients’ health system interactions and prescription dispensations. When asked about providing care virtually, participants reported utilizing these resources to help determine whether it was appropriate to renew a prescription virtually: “*We’re using PharmaNet a lot. And it’s like, ‘Oh, yeah, they picked up within the last five days, so it’s safe’”* (FPOUD01). These system-wide resources also aided FPs’ management of OUD patients between virtual appointments to ensure quality of care is not sacrificed:

*… a lot of the care that I do is outside of patient interactions. I manage patients a lot without seeing them even, or talking to them, through the collateral resources, and through other information that I have, because a lot of them aren’t able to engage in traditional structure of healthcare interactions. And we can do a lot knowing what they’ve been picking up, how often they’ve been picking up, what their dose is, what their [life skills worker] or the nurse or whatever got from them. So I’m able to do a ton of care. And that way, that’s I think actually fairly high-quality care. And a lot of that is virtual, too, like with other providers. So I’m talking to the nurse on the phone, I’m talking to the pharmacist on the phone, and I’m pulling up their Pharmanet, and I’m looking at their CareConnect, like their previous lab results and stuff like that. I would say actually, relative to other populations, I do a ton of care outside of the patient being present.* (FPOUD10)

Communication with an interdisciplinary team, such as nurses and pharmacists, who may interact with patients more frequently than physicians themselves, provided reassurance to physicians who were prescribing OAT virtually. This was particularly the case where FPs had not connected with the patient recently. In fact, when asked about supports or resources that helped them deliver virtual primary care, most physicians mentioned the valuable support of external health professionals, as they provided on-the-ground insight on the patient’s situation:

*But our pharmacy team, we've got a very amazing team that will actually go out in the community and deliver the OAT to patients. […] They're contacting patients pretty much every day. Even if I can't talk to them, I can call the pharmacist to say, ‘How was [patient] today when you saw him?’* (FPOUD09)

Reliance on an interdisciplinary team is particularly important for physicians who provide OUD care remotely to PWOUD living in rural communities, as it allows for more comprehensive and feasible care:

*If you have a nurse that’s present in a community… I’m thinking of like, you know, a rural and remote Indigenous community, and you have a skilled remote nurse who’s there, and is your eyes and ears into the clinical situation, and is experienced and skilled to be able to fill you in on those details […] I would feel far more comfortable doing that if there was an allied health professional that I had a trusted relationship with.* (FPOUD08)

Support from, and collaboration with, community-based social service staff such as life skills workers and shelter or transition housing staff, also plays an important role in facilitating effective virtual care. These individuals help bridge gaps for patients without reliable access to a private phone and assist with in-person components that enhance physicians’ willingness in providing care remotely.

*We are so lucky to have life skills workers. That’s a huge part. And that’s them sometimes helping patients go for tests that we’re ordering or making it in when I do want to see them in person. So, life skills workers are a huge resource for virtual care as well.* (FPOUD10)

Ultimately, interdisciplinary collaboration across the various settings and between the different people engaged in patients’ treatment supports continuity of care and enhances physicians’ willingness in delivering treatment: “*The interdisciplinary [team] makes it much easier and gives you a lot more comfort*” (FPOUD06).

Finally, when participants were asked about instances in which they prescribed OAT virtually, some participants reported that turning to specialized medical support networks for guidance was particularly helpful. These provincial networks include the BC Centre on Substance Use (BCCSU) helpline, which provides addiction medicine support to healthcare providers (48); BC Pathways, an online resource offering referral information and access to medical and community service directories (49); and the Rapid Access to Consultative Expertise (RACE), a program that connects healthcare professionals with specialists for urgent consultations (50):

*I know the BCCSU has a 24/7 line as well. But to be honest, I've tended to always use RACE […] once addictions [specialists] came on for them, it was kind of my go-to, easy-to-use one. I do feel like we're well supported between that and the BCCSU line. And the doctors that answer are excellent*. (FPOUD01)

As described by a participant without formal addiction training, access to external addiction medicine resources enabled OAT delivery in a way that would not have been possible without access to consultation resources: “*I actually phoned the addictions physician on call […] this was before I had done the POATS-P course. ‘Ah, am I even allowed to prescribe Suboxone? I do not know.’ So, I called them, and they told me what to do.”* (FPOUD04).

Other providers felt that external supports were insufficient to make them feel comfortable to prescribe OAT virtually, leading them to refer patients to other care services and care settings: “*I did refer informally people to the opioid agonist team to get started because I could not do it - I did not feel I could do an induction virtually*” (FPOUD02).

## 4 Discussion

This paper explored primary care physician perspectives on virtual OAT prescribing. Through a hybrid inductive-deductive thematic analysis, we inductively identified themes which we then deductively organized using Ross et al.’s (45) Barriers and Facilitators framework. Our results reflect multi-level influences shaping physician’s decisions to provide OUD care virtually. Patient-level factors include individual patients’ OAT stage, comorbidities, type of OAT medication, and environmental triggers. Physician-level facilitators include pre-existing relationships between the patient and the physicians or clinic, and physician’s perception of a patient’s stability. Finally, health system-level facilitators include the use of centralized electronic data systems, providing care alongside an interdisciplinary team, and access to provincial specialized medical support networks. In each case, the absence or opposite of these conditions posed barriers to physicians’ willingness to prescribe OAT virtually.

In our sample, physicians reported a general reluctance to initiate OAT virtually. This finding is consistent with existing literature, which shows that most physicians prefer in-person appointments for OAT inductions, while virtual care is more commonly endorsed for other phases of OAT (i.e., stabilization and retention) (51–53). This may be tied to the current evolving practice standards around prescribing OAT virtually, which request FPs to either have a longitudinal relationship with the patient, or perform and document a comprehensive in-person or virtual assessment and be available to take on the patient’s OUD management long-term (54).

Physicians we interviewed often preferred in-person visits for patients who presented with complex clinical profiles, including comorbid conditions such as concurrent psychiatric disorders. Similar research suggests that PWOUD with comorbid depression or anxiety were more likely to receive virtual care, while those with polysubstance use or late-term pregnancy were more often seen in person (55, 56). Collectively, these findings show that the nature of the comorbid conditions and provider perceptions of clinical complexity may influence physicians’ discretion to prescribe OAT virtually.

Participants working in neighborhoods with concentrations of visible substance use indicated a willingness to support PWOUD who had left and sought to avoid returning to such high-risk environments, recognizing that requiring in-person appointments was counterproductive while virtual appointments could help protect individuals’ trajectories. The availability of take-home doses further supports this flexibility, patient agency, and treatment retention (57) without daily exposure to high-risk community settings. During the COVID-19 pandemic, federal exemptions and interim treatment guidance on OAT enrollment allowed for increased access to take-home doses (58). Research showed that these guidelines led to an initial increase in take-home dosing; however, this prescribing pattern was not sustained over time (59, 60). Consequently, PWOUD continue to face requirements that place the onus on them to “earn” the right to take-home doses (31). Efforts should focus on the implementation of a minimally disruptive medicine approach that recognizes structural barriers to in-person OUD care and enhances engagement with the healthcare system. In turn, consistent engagement may increase physicians’ willingness to deliver OUD care that is flexible, person-centered, and responsive to individual needs.

Pre-existing patient–provider or patient–clinic relationships were frequently cited as shaping physicians’ willingness to prescribe OAT virtually in our interviews. This finding is consistent with previous studies reporting physician hesitation in providing OAT virtually with patients they have not previously treated in-person due to concerns about risk, safety, and liability (51, 53). This underscores the importance of ensuring continuity of care by pairing patients with consistent primary care providers, particularly as virtual modalities may challenge relationship-building and the development of trust over time (61, 62). Nonetheless, our participants reported that in instances where prescribing OAT was the only viable path to maintaining continuity of care, they adopted a harm reduction approach of meeting patients where they are. This aligns with existing evidence indicating that harm reduction strategies are associated with improved treatment engagement, reduced overdose risk, long-term adherence and better overall health outcomes among PWOUD (63, 64).

A physician’s perception of patient stability may be an important factor in their willingness to prescribe OAT virtually. Our findings suggest that definitions of “stability” broadened during the COVID-19 pandemic, with some physicians increasingly considering social determinants of health (e.g., housing stability, employment) as relevant indicators in addition to traditional clinical measures (e.g., urine drug screen). The use of virtual care is associated with improved treatment retention compared to in-person care alone (33, 65). Therefore, restricting virtual OAT access to only those patients whom clinicians deem “stable” may inadvertently reinforce structural barriers to care. Existing research supports this, with racialized, rural, and lower-income individuals less likely to receive OAT care virtually, sometimes in spite of patients’ preference for virtual appointments (51, 61, 66–68), thereby limiting access for PWOUD who may benefit most from flexible, low-barrier treatment models (69). These insights underscore the importance of future research exploring access to virtual OAT among marginalized populations in Canada.

Our participants identified team-based care models and formal integration between primary care and other healthcare and social services in supporting family physicians’ provision of OUD-specific care. Social workers, nurses, and pharmacists play complementary roles in supporting FPs to deliver OUD care virtually. For example, social workers address social determinants of health (e.g., housing insecurity, technology access) that can otherwise limit patients’ engagement, while coordinating with community organizations to facilitate continuity of care (70, 71). Pharmacists monitor adherence and share treatment insights to guide safe prescribing (72). The continuous virtual presence of nurse care managers (73), in-person peer support services (74), and community outreach (75) contributes to engagement in OAT programs and health services utilization, showing that these professionals help foster the stability indicators that physicians often rely on when deciding whether to prescribe OAT virtually. Together, these professionals help sustain treatment retention and reduce barriers to virtual care, particularly in rural and underserved areas (55, 76, 77).

Finally, physicians heavily relied on specialized medical support networks and resources when providing OAT virtually, showing the importance of continuing to sustain accessible, provider-to-provider support systems to promote equitable uptake of OUD care delivered virtually across clinical settings (52).

Our findings build upon those of Ross et al. (45), who identified multi-level barriers and facilitators to primary care access for individuals with mental health and/or substance use issues. Both studies underscore the importance of patient-provider relationships, with our results suggesting that prior in-person contact is often a prerequisite for physician willingness to initiate OAT virtually. At the system level, both studies also support the value of interdisciplinary, collaborative models; our work further illustrates how provider resources (e.g., specialized medical support networks) uniquely empower physicians to provide care.

A key strength of this study is the use of a robust inductive-deductive hybrid thematic analysis, which allowed themes to emerge organically without constraint, while anchoring them in an existing framework *post-hoc* to ensure analytic coherence. Nevertheless, our study has limitations. A majority of the participants were women practicing in urban areas, which many not be representative of the broader population of BC physicians who provide OUD care. Many family physicians in our sample reported that PWOUD comprised a high proportion of their patient panel, showing substantial experience in providing OUD management. Although this aspect led to rich reflections on providing OUD management and reinforces the credibility of the findings, it may also limit transferability to other FPs who support fewer PWOUD in their practices (78). Additionally, this sample was limited to FPs practicing in BC, a province known for its progressive policies around OUD care and means to support the delivery of virtual care. Consequently, the findings may not be generalizable or transferable to jurisdictions with different approaches to OAT and/or less established virtual care system (79, 80).

## Data Availability

The datasets presented in this article are not readily available because the interview transcripts may include personal identifiable information. Requests to access the datasets should be directed to LH, lindsay_hedden@sfu.ca.
